# Anti-neuroinflammatory Potential of Natural Products in Attenuation of Alzheimer's Disease

**DOI:** 10.3389/fphar.2018.00548

**Published:** 2018-05-29

**Authors:** Bushra Shal, Wei Ding, Hussain Ali, Yeong S. Kim, Salman Khan

**Affiliations:** ^1^Department of Pharmacy, Faculty of Biological Sciences, Quaid-i-Azam University, Islamabad, Pakistan; ^2^Department of Neurosurgery, Rizhao Hospital of Traditional Chinese Medicine, Rizhao, China; ^3^College of Pharmacy, Seoul National University, Seoul, South Korea

**Keywords:** Alzheimer's disease, neuroinflammation, natural products, herbal formulation, phytochemicals, neuroprotection

## Abstract

Alzheimer's disease (AD) is a chronic progressive neurodegenerative disorder associated with dementia and cognitive impairment most common in elderly population. Various pathophysiological mechanisms have been proposed by numerous researcher, although, exact mechanism is not yet elucidated. Several studies have been indicated that neuroinflammation associated with deposition of amyloid- beta (Aβ) in brain is a major hallmark toward the pathology of neurodegenerative diseases. So, there is a need to unravel the link of inflammatory process in neurodegeneration. Increased microglial activation, expression of cytokines, reactive oxygen species (ROS), and nuclear factor kappa B (NF-κB) participate in inflammatory process of AD. This review mainly concentrates on involvement of neuroinflammation and the molecular mechanisms adapted by various natural compounds, phytochemicals and herbal formulations in various signaling pathways involved in neuroprotection. Currently, pharmacologically active natural products, having anti-neuroinflammatory potential are being focused which makes them potential candidate to cure AD. A number of preclinical and clinical trials have been done on nutritional and botanical agents. Analysis of anti-inflammatory and neuroprotective phytochemicals such as terpenoids, phenolic derivatives, alkaloids, glycosides, and steroidal saponins displays therapeutic potential toward amelioration and prevention of devastating neurodegeneration observed in AD.

## Introduction

Alzheimer's disease (AD) is a neurodegenerative disorder that accounts for age-related dementia in more than 80% cases worldwide (Anand et al., 2013). It is a progressive disease leading to disturbances of memory and cognitive function. It is estimated that ~5 million people with age 65 years or older and 200,000 people younger than 65 years are affected by AD. However, the total estimated prevalence by 2050 is expected to be 13.8 million (Alzheimer's Association, 2015). Limitation of existing preventive methods has increased the importance of intervention therapies using natural products rich in antioxidant and flavonoid content. The neuroprotective effect of natural compounds has been explored through preclinical and clinical studies using *in vitro* and *in vivo* models (Essa et al., 2012). Currently, approved treatments by US Food and Drug Administration (FDA), includes acetylcholinesterase inhibitors (AChEIs) and N-Methyl-D-Aspartate (NMDA) receptor antagonists that are involved in the symptomatic treatment of AD (Auld et al., 2002). However, due to serious side effects and limitations these drugs are rarely prescribed (Kumar and Singh, 2015). These are forms of palliative care, which slows the progression of cognitive symptoms and prevents any worsening of the patient's symptoms (Farlow et al., 2008). There is no proper treatment leading to cure AD till now (Ramirez-Bermudez, 2012). Immense efforts are directed toward identification of various disease-modifying therapies and discovering drugs targeting molecular pathways and blocking progression of AD (Kurz and Perneczky, 2011).

**Figure 1:**
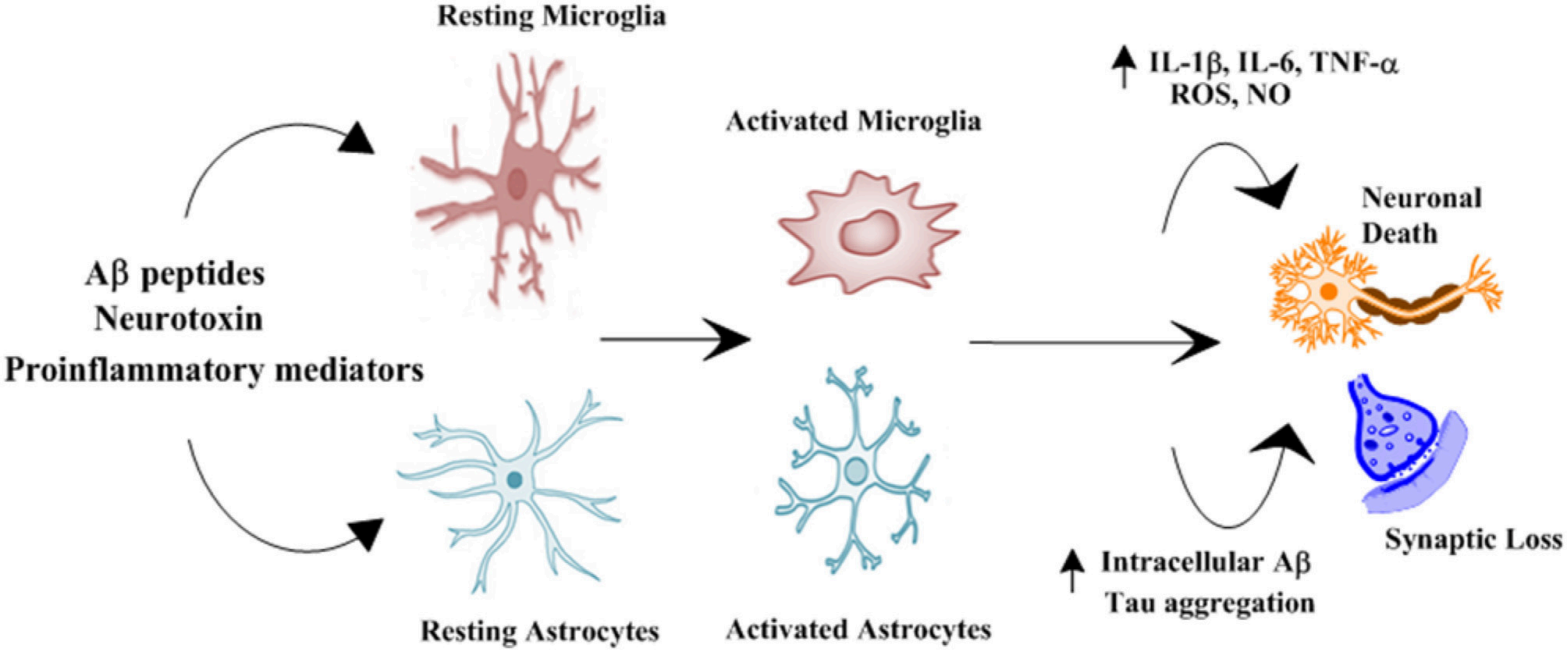
Schematic representation of role of glial cells in pathophysiology of Alzheimer's disease. Numerous stimuli such as Aβ peptides, neurotoxin and proinflammatory mediators activates microglial cells and astrocytes. Activated microglial cells and astrocytes results in increased production of proinflammatory cytokines and intracellular Aβ and Tau aggregation resulting in synaptic loss and neuronal death (Morales et al., 2014).

Multiple biological processes such as cognitive decline, abnormal deposition of amyloid β peptide (Aβ), accumulation of neurofibrillary tangles (NFTs), neuroinflammation, depletion or insufficient synthesis of neurotransmitters, oxidative stress, and abnormal ubiquitination linked to neurodegenerative diseases such as AD (Korolev, 2014). Genetic and environmental factors both contribute to the pathogenesis of the AD. On the basis of a number of causative factors several hypotheses have been presented to explain this multifactorial disorder, this includes the Aβ hypothesis, tau hypothesis, cholinergic hypothesis, and inflammation hypothesis (Rashid and Ansari, 2014). Recently inflammation hypothesis has gained considerable importance, innate immunity and neuroinflammation is involved in pathogenesis of neurodegenerative processes such as Alzheimer's disease (AD) due to the production of pro-inflammatory cytokines influencing the surrounding brain tissue, shown in Figure 1 (Tan et al., 2013). Immune cells such as microglia activate resulting in production and release of proinflammatory cytokines, such as IFN-γ, IL-1β, and TNF-α (Tan et al., 2013). These cytokines stimulate the nearby astrocyte–neuron to produce further amounts of Aβ42 oligomers, thus, activating more Aβ42 production and dispersal (Dal Prà et al., 2015). Increase in the level of pro-inflammatory cytokines has been observed in the brain, serum, and cerebrospinal fluid (CSF) of AD patients in previous reports (Dursun et al., 2015). In AD Aβ form insoluble and extracellular pathological aggregates which attract microglial cells, forming clusters of microglia at sites of Aβ deposition (Streit et al., 2004). Experimental studies in animals supported the idea of involvement of microglia in phagocytosis and degradation of amyloid, such phagocytosis is ineffective in AD (Weldon et al., 1998). Activation of microglial cells and neuronal loss have been reported by direct injection of Aβ into the brain (Weldon et al., 1998). TNF-α and IL-1β affects the function of blood-brain barrier, as well as also leads to the activation of astrocytes as a secondary response, long-term effect of these cytokines can be detrimental to the astrocyte survival (Lim et al., 2013). However, it reveals a potential new target cell explaining negative effects of cytokines on brain tissue during neuroinflammation (Lim et al., 2013). Many studies have also directly associated cognitive decline with the levels of cytokines in AD patients at all stages, however, there are no drugs approved for neuroinflammation in AD (Garcez et al., 2017).

Natural polyphenolic phytochemicals have recently gained greater attention as alternative therapeutic agents against AD (Essa et al., 2012). They are considered less toxic and more effective than novel synthetic drugs (Kim et al., 2010). However, commonly herbal medicines are prepared from the crude materials, which raise questions regarding their mechanism of action and medicinal effects. Recently, research has been focused on specific active components rather than on an entire herb (Morales et al., 2014). Therefore, there is a need to identify a number of active constituents and to characterize them according to their therapeutic potentials, focusing on their effects toward neurodegenerative diseases such as AD (Kim et al., 2010). This review focuses on the natural products and their derivatives that are involved in regulation of inflammatory pathways to treat AD. Sufficient evidence suggests the role of phytochemicals in prevention and treatment of AD by targeting neuroinflammatory pathway.

## Neuroinflammation and signaling cascades in Alzheimer's disease

### NF-κB pathway in Alzheimer's disease

**Figure 2:**
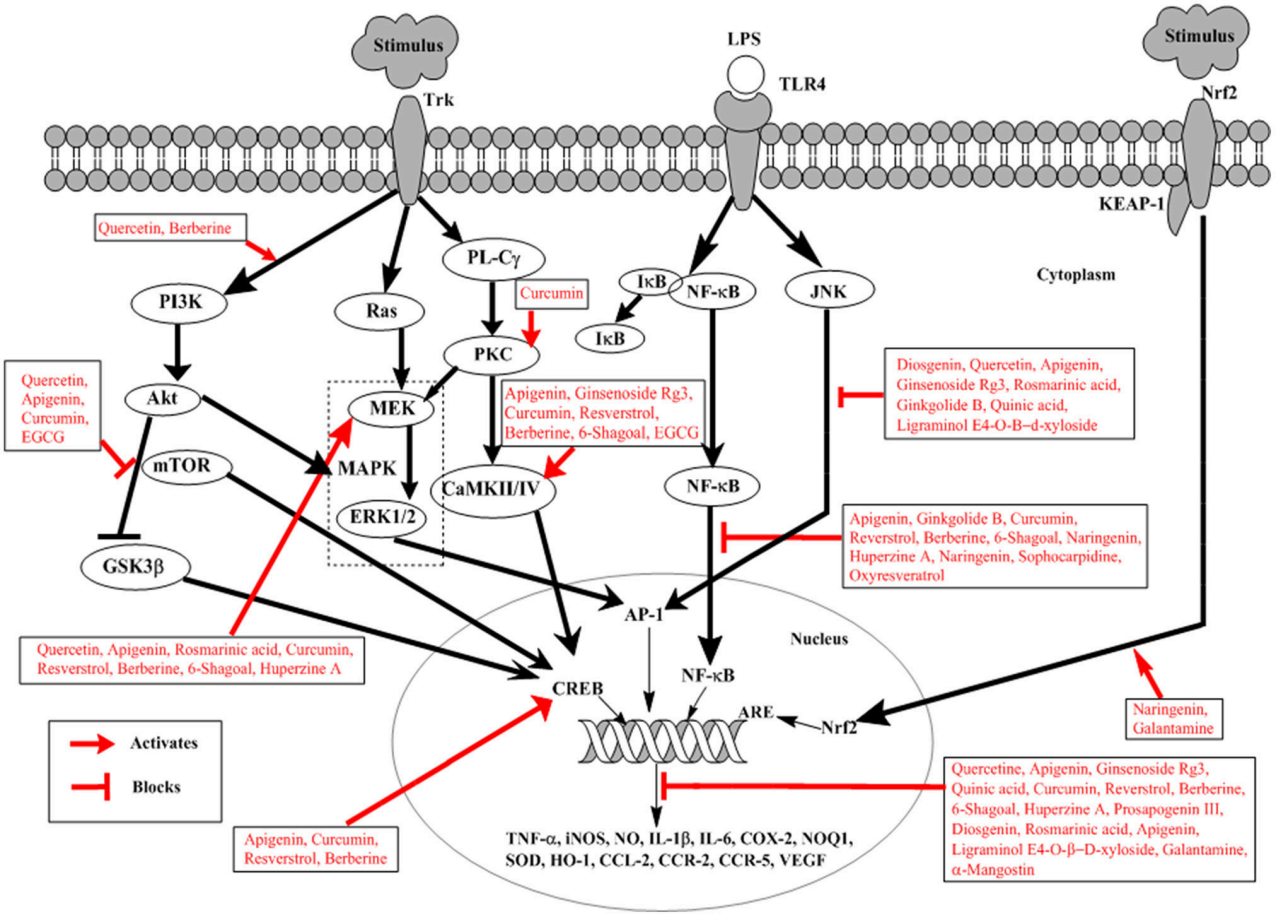
Schematic representation of natural products involved in neuroprotection against Alzheimer's disease. External stimulus binds to Trk receptor activating PI3K/AKT, Ras/MAPK, and PL-Cγ pathways. LPS binds to TLRs activating NF-κB and JNK signaling. External stimulus releases Nrf2 from Nrf2-KEAP-1 complex and activates ARE. Trk, tyrosine kinase receptor; LPS, lipopolysaccharide; TLRs, toll-like receptors; PI3K, phosphatidylinsoitol-3-kinase; MAPK, mitogen-activated protein kinase; mTOR, mammalian target of rapamycin; ERK, extracellular signal-regulated kinases; Nrf2, nuclear factor e2-related factor 2; KEAP-1, Kelch-like ECH-associated protein-1; MEK, mitogen-activated protein kinase; PL-Cγ, phospholipase Cγ; NF-κB, nuclear factor-kappa B; PKC, protein kinase C; JNK, c-Jun N-terminal kinase; ARE, Antioxidant response element; CREB, cyclic adenosine monophosphate response element binding protein; IκB, inhibitory kappa B; CaMKII/IV, Ca2+-calmodulin kinase II/IV; GSK3β, glucose synthase kinase-3β. Diosgenin, Prosapogenin III, Quercetin, Apigenin, Ginsenoside Rg3, Rosmarinic acid, Ginkgolide B, Limonoid, Quinic acid, Curcumin, Resverstrol, Berberine, 6- Shagoal, Ligraminol E4-*O*-β-d-xyloside, Huperzine A, Sophocarpidine, Naringenin, Epigallocatechin-3-galate (EGCG), Oxyresveratrol, α-Mangostin, Galantamine.

The nuclear factor-kappa B (NF-κB) is a best-characterized transcription factor, expressed ubiquitously and regulating the expression of many genes, responsible for encoding proteins involved in the processes of inflammation and immunity as shown in Figure 2 (Li and Verma, 2002). Apart of these roles NF-κB is shown to be involved in brain function, particularly in neurodegenerative diseases like AD (O'neill and Kaltschmidt, 1997). Aβ induced neurotoxicity has been linked to NF-κB activation (Longpré et al., 2006). Neuronal and microglial cells when treated with Aβ results in activation of NF-κB signaling (Longpré et al., 2006). In brains of AD patients, NF-κB activation has been detected (Boissière et al., 1997). Use of long-term anti-inflammatory drugs has shown to suppress the progression and onset of AD indicating a close relation between NF-κB and pathogenesis of AD (Hong, 2017). Therefore, potential therapeutic approach against AD can involve the modulation of Aβ-induced activation of NF-κB signaling.

### Akt/PI3K pathway in Alzheimer's disease

The Akt/PI3K pathway is involved in survival, proliferation, growth, and migration of cells (Yu and Koh, 2017). Akt is an important and direct effectors of PI3K/Akt pathway; reportedly involved in many different substrates activation in cellular signaling as shown in Figure 2 (Yu and Koh, 2017). Among them glycogen synthase kinase (GSK)-3β is important and well-known involved in direct induction of tau phosphorylation. GSK-3β at serine 9 when phosphorylated by activated Akt, results in inhibition of GSK-3β (Plyte et al., 1992). In AD GSK-3β phosphorylates tau protein thus inducing detachment of tau proteins from microtubules, which aggregates with each other. This causes the loss of function of microtubule, thus increasing vulnerability of cells and inducing cell death (Yu and Koh, 2017). In addition Akt also activates mTOR, whose signaling is closely related to the presence of soluble amyloid beta (Aβ) and tau protein. Injecting Aβ oligomers into the hippocampus of normal mice has shown mTOR hyperactivation (Caccamo et al., 2011). In AD mTOR signaling pathway is considered to be one of the mechanisms involved in Aβ-induced toxicity (Lafay-Chebassier et al., 2005). Increased Aβ concentration increases mTOR signaling, however, large concentration of cytotoxic Aβ decreases mTOR signaling (Lafay-Chebassier et al., 2005). This role of mTOR signaling is controversial in amyloid hypothesis.

### MAPK pathways in Alzheimer's disease

MAPK plays an important role in neuronal survival and death *in vitro* and in brain by signaling oxidative stress and cell cycle control as shown in Figure 2 (Zhu et al., 2000). Various studies have shown the increased level of active ERK in AD (Zhu et al., 2001a). Thus, resulting in impaired hippocampus function and contribute toward memory impairment (Zhu et al., 2001a). JNK has shown to have role in AD pathogenesis; it co-localizes with DNA damage and is associated with neurofibrillary pathology (Smith et al., 2000). Since the oxidative stress has a well-documented role in AD, so it is suspected that JNK pathway is activated as a response to cellular stress in AD (Smith et al., 2000). Increased level associated with the pathology of NFTs and senile plaque in AD brain (Zhu et al., 2000). Further evidence has been provided convincing the abnormally activated p38 pathway in AD because of the increased activation of MKK6 as an immediate upstream activator of p38 (Zhu et al., 2001b). The activated MKK6-p38 is more prominent in neurons than in microglia, suggesting direct contribution toward degeneration of neurons in AD (Zhu et al., 2001b). However, simultaneous activation of ERK and JNK has a high tendency to develop AD representing one of the initial events in disease pathogenesis likely precipitating further alterations (Zhu et al., 2001a).

### Nrf2 pathway in Alzheimer's disease

Nuclear factor E2-related factor 2 (Nrf2) is the transcription factor which activates expression of the genes with antioxidant activity translocating in response to oxidative stress from the cytoplasm into the nucleus as indicated in Figure 2 (Itoh et al., 1997). It is an attractive therapeutic target for the prevention of AD (Kerr et al., 2017). Antioxidant response element (ARE) is a common response element of the genes involved in the reduction of oxidative stress, inflammation and accumulation of toxic metabolites. As a result of oxidative damage in AD, there is an upregulation of Nrf2 expressions in the neuron. In AD, there is a reduction in the levels of some ARE-containing gene products, suggesting disruption of the pathway (Itoh et al., 1997). Moreover, the *in-vivo* evidence also suggests that inhibition of Keap 1, prevents the neuronal toxicity caused by Aβ42 peptides initiating AD, correlating with activation of Nrf2 (Kerr et al., 2017).

### CREB signaling in Alzheimer's disease

Cyclic-AMP response element binding protein (CREB) has been known to be important in memory formation (Kandel, 2012). Studies propose dysfunctions in the CREB signaling in various mouse models of AD (Bartolotti et al., 2016). It has been shown that CREB and pCREB its activated form, along with CREB-binding protein (CBP) the transcription cofactors and p300 are reduced in prefrontal cortex of AD, indicating dysfunctional CREB signaling in AD (Bartolotti et al., 2016).

### Potential role of natural products in treatment of Alzheimer's disease

Despite the accumulation of valuable knowledge and incredible innovation of modern medicine there is no effective cure of world's most problematic diseases such as Alzheimer Disease (Rasool et al., 2014). Synthetic drugs are useful for managing these diseases, however, they still possesses severe side effects (Rasool et al., 2014). Due to the increase need of novel and effective treatment, natural products have gained attention as promising therapeutic agents recently neuroprotective treatments have demonstrated that animal based products such as omega-3, fatty acids, and plant based compounds inhibit cellular toxicity and shows anti-inflammatory effects (Wollen, 2010). Due to the potential of anti-inflammatory, antioxidant and neuroprotective properties of phytochemicals with minimal side effects have revealed the potential to improve and prevent neurodegeneration in AD (Cooper and Ma, 2017). It is speculated that onset and progression of AD might be lowered by traditional herbs and phytochemicals by targeting multiple pathological targets (Venkatesan et al., 2015). Herbs are found to be involved in regulation of mitochondrial stress; free radical scavenging systems, apoptotic factors, and neurotrophic factors (Starkov and Beal, 2008). Progression of AD is also accelerated by inflammation contributing to neurodegeneration (Cooper and Ma, 2017). Therefore, early prevention and management of inflammation might serve as a potential treatment or reducing symptoms of AD (Cooper and Ma, 2017).

Phytochemicals having anti-inflammatory, antioxidant and anti-amyloid activity can interact with neuroinflammatory mediators. The evidence of blood brain permeability of various natural products is still not conclusive. However, history of their traditional use and abundant data from animal studies demonstrates their ability to penetrate the blood brain barrier (BBB) (Kam et al., 2012). After an oral administration, principle metabolites of (–)-epicatechin have been detected in rodent brain (Van Praag et al., 2007). Similar results were also observed with epigallocatechin-3-galate (EGCG) (Lin et al., 2007) and *Ginko biloba* extract (Rangel-Ordóñez et al., 2010). It was observed that the flavonoids from *G. biloba* extract were distributed in the hippocampus, striatum, prefrontal cortex and cerebellum (Rangel-Ordóñez et al., 2010). The ability of metabolites to penetrate the BBB depends upon the degree of lipophilicity, polarity, and molecular weight of each compound (Rajadhyaksha et al., 2011). Less polar lipophylic compounds with molecular weight <500 daltons are easily permeable (Rajadhyaksha et al., 2011). The brain entry also depends on the ability to interact with specific efflux transporters expressed in BBB which includes P-glycoprotein, as observed in quercetin and naringenin flux into the brain (Youdim et al., 2004). Furthermore, the time-dependent transport studies across cerebral capillary endothelial cells of quercetin and catechin suggest the capability of BBB transfer (Faria et al., 2010). These studies demonstrate the ability of natural products to reach the areas of brain involved in neurodegenerative disease. Despite this, further investigation regarding extent of brain bioavailability of natural compounds can be performed before any firm conclusion.

### Neuroprotective effect of steroid phytochemicals in Alzheimer's disease

#### Diosgenin

**Figure 3:**
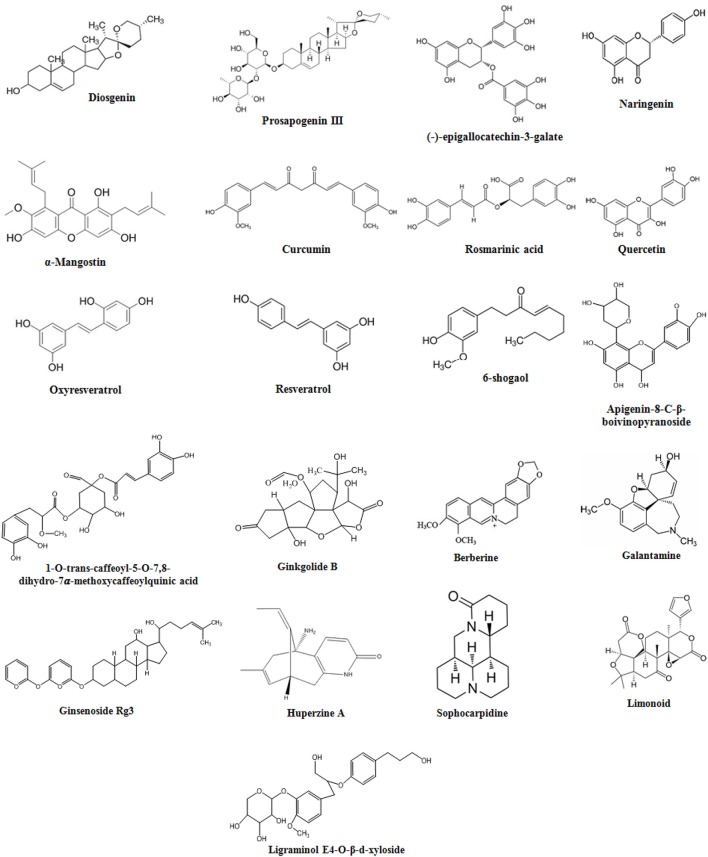
Chemical Structures of natural compounds.

Diosgenin is a saponin aglycon a chemical constituent of *Dioscorea nipponica* (Table 1), chemical structure in Figure 3. It is also found in numerous other plants such as *Dioscorea villosa, Trigonella foenum graecum, Solanum xanthocarpum, S. incanum Lloydia*, and *Costus speciosus*. It has a wide variety of reported functions, which includes its anticancer, antifungal, and anti-inflammatory properties (Chiu and Lin, 2008). A previous report has shown anti-inflammatory effect of saponin aglycons, including diosgenin on LPS-stimulated RAW 264.7 macrophages (Chiu and Lin, 2008). Furthermore, it regulates the activity of NF-kB (Shishodia and Aggarwal, 2006), lipoxygenase (Nappez et al., 1995), and cyclooxygenase-2 (Moalic et al., 2001), also antagonizes the CXCR3 chemokine receptor involved in mediation of inflammatory responses (Ondeyka et al., 2005). Diosgenin dose dependently suppresses the production of inflammatory factors like nitric oxide (NO), monocyte chemoattractant protein-1 (MCP-1), and tumor necrosis factor-α (TNF-α) in the case of coculture of macrophages and adipocytes (Hirai et al., 2010). The nitrogen analog of diosgenin, tomatidine a steroid saponin has been reported to inhibit NF-kB and JNK signaling acting as an anti-inflammatory agent (Chiu and Lin, 2008). Consistent with these reports, it is suggested that in macrophages FFA-induced inflammation is inhibited by diosgenin through suppression of both JNK/AP-1 and IkB/NF-kB signaling pathways (Hirai et al., 2010). Therefore, through various studies it has been suggested that diosgenin has anti-neuroinflammatory potential against neurodegenerative diseases such as AD.

**Table 1:** Steroid phytochemicals that affect Alzheimer's disease.

**Plant source**	**Phytochemicals**	**Pharmacological effects**	**Medicinal use**	**References**
**STEROID PHYTOCHEMICALS**				
*Dioscorea nipponica*	Diosgenin	Inhibits IκB/ NF-κB pathway and MAPK pathways; ERK, p38, and JNK	Neuroinflammation, and neuroprotection	Hirai et al., 2010
*Liriope platyphylla*	Prosapogenin III	Blocks MAPK/NF-κB pathway, inhibiting release of inflammatory mediators	Anti-inflammation, antioxidant, and neuriotogenic properties	Han et al., 2013

#### Prosapogenin III

Prosapogenin III (Table 1), a steroidal saponin extracted from roots of *Liriope platyphylla* (Liriopis Tuber). It is a medicinal plant reported to possess anti-diabetes, anti-asthma, neuriotogenic and anti-inflammatory properties (Han et al., 2013). Effect of prosapogenin III (Figure 3) was evaluated in LPS-induced RAW264.7 cells expressing inflammatory mediators such as anti-inflammatory cytokines, inducible nitric oxide (iNOS), nitric oxide (NO), and cyclooxygenase-2 (COX-2), also interleukin-1β (IL-1β), and IL-6. Results obtained from the study indicated its anti-inflammatory effects in activated macrophages by blocking MAPK/NF-κB signaling inhibiting production of inflammatory mediators (Han et al., 2013). For investigation of neuroprotective effect of *Liriope platyphylla* extract (LPE), its effect was evaluated in SH-SY5Y human neuroblastoma cells having hydrogen peroxide (H_2_O_2_)-induced injury (Park et al., 2015). The results demonstrated that LPE has neuroprotective effects by protecting cell growth through inhibition of p38 phosphorylation in a H_2_O_2_-induced stressful background (Park et al., 2015). Therefore, prosapogenin III might be a potential candidate for the amelioration of AD.

### Neuroprotective effect of phenolic phytochemicals in Alzheimer's disease

#### Quercetin

Quercetin, a flavonoid is derived from mulberry fruit *Morus alba* (Table 2) from family Moraceae (Dajas, 2012). This fruit contains palmitic acid, linoleic acid, vitamin C, phenolics, oleic acid, gallic acid, anthocyanins, and quercetin having anti-inflammatory, anti-carcinogenic, and antioxidant properties (Dajas, 2012). Due to its anti-inflammatory property, it has been studied as a lead compound showing neuroprotective effect in the animal model of neurodegeneration (Bahar et al., 2017). Quercetin (Figure 3) is a strong inhibitor of both 5-lipoxygenase (5-LOX) and cyclooxygenase-2 (COX-2) enzymes involved in production of eicosanoids from arachidonic acid and also has inhibitory action on prostaglandins (PG) production and NF- κB activation (Pany et al., 2014). Quercetin enhance neuroprotection through their antioxidant property by scavenging of free radicals (Kim et al., 1999). It easily crosses the blood brain barrier (Chen et al., 2009). It has also been shown that in the brains of Sprague-Dawley (SD) rats and SK-N-MC cell lines of human neuroblastoma quercetin could inhibit apoptosis and inflammatory response induced by manganese (Mn) by activating HO-1/Nrf2 and inhibiting NF-κB signaling (Bahar et al., 2017). These studies suggest the neuroprotective property of quercetin and various other extracts of *M. alba* against the AD.

**Table 2:** Phenolic phytochemicals that affect Alzheimer's disease.

**Plant source**	**Phytochemicals**	**Pharmacological effects**	**Medicinal use**	**References**
**PHENOLIC PHYTOCHEMICALS**				
*Camellia sinensis*	Epigallocatechin-3-galate (EGCG)	Inhibits MAPK and NF-κB activation, attenuates IL-1, IL-6, IL-8, COX-2 and PGE_2_ production. Also induces BDNF, NGF secretion, and inhibits cas3 and ROS level	Neuroinflammation, neuroprotection, and cognitive deficit	Kim et al., 2007; Liu et al., 2014
*Cirus paradise and Citrus sinensis*	Naringenin	Activating Nrf2/ARE signaling, upregulates antioxidant enzymes, decreases NO, cytokines and NF-κB signaling	Anti-inflammatory, antioxidant and neuroprotective	Raza et al., 2013; Lou et al., 2014
*Curcuma longa*	Curcumin	Activates PKC/ERK-mediated CREB regulation and Akt/GSK-3β mediated regulation, induces BDNF secretion, and inhibits Cas3, TNF-α, and NF-κB levels	Neuritogenesis, neuroinflammation, and anti-oxidant properties	Hoppe et al., 2013; Nam et al., 2014
*Garcinia mangostana* *Melissa officinalis*	α-Mangostin Rosmarinic acid	Suppresses inflammation, increases BDNF level and decreasing phospho-tau (p-tau) Suppresses the expression of HIF-α, IL-1β, TNF-α and caspase 3	Alzheimer's Disease Neuroprotective, antioxidant properties	Huang et al., 2014 Bayat et al., 2012
*Morus alba*	Quercetin	Inhibits COX-2, 5-LOX enzymes and GSK-3β in PI3K pathway and inhibits NF-κB activation, also is involved in free radical scavenging	Anti-inflammatory and antioxidant properties	Kimata et al., 2000; Pany et al., 2014
*Morus alba L*.	Oxyresveratrol	Decreases TNF-α, IL-6 and inhibits activation of NALP3, caspase-1, NF-κB and inhibits phosphorylation of ERK, c-JNK and p38	Anti-inflammatory and anti-apoptotic effects	Chung et al., 2003; Wang et al., 2014
*Passiflora edulis and P. alata*	Apigenin-8-C-β-digitoxopyranoside, luteolin-8-C-β-boivinopyranoside, and apigenin-8-C-β-boivinopyranoside	Inhibits NO and PGE2 production, suppresses expression of ERK 1/2, p38, MAPK, JNK and COX-2	Anti-AD, and anti-inflammatory properties	Choi et al., 2014
*Pimpinella brachycarpa*	3,5-O-trans-dicaffeoylquinic acid methyl ester, 1-O-trans-caffeoyl-5-O-7,8-dihydro-7α-methoxycaffeoylquinic acid	Inhibits NO, iNOS production, and boost antioxidant system	Antioxidant, anti-inflammatory, and neuroinflammation	Soh et al., 2003; Lee et al., 2013
*Vitis vinifera*	Resveratrol	Inhibits synthesis and release of pro-inflammatory mediators, inhibits iNOS, COX-2, NF-κB, AP-1 and promotes anti-inflammatory molecules IL-10	Antioxidant, anti-inflammatory, and neurodegenerative disorders	Song et al., 2014
*Zingiber officinale*	6-shogaol	Induces NGF, BDNF, and GDNF secretion, inhibiting NO, TNF-α, IL-1β, p38, Bax, NF-κB, iNOS, PGE2, and ROS level and increases Bcl-2, and SOD levels.	Anti-inflammatory, and antioxidant	Ha et al., 2012

#### Oxyresveratrol

Oxyresveratrol, a major stilbene of white mulberry *M. alba* (Table 2), family Moraceae showed interesting anti-inflammatory effects (Chung et al., 2003). It has shown to inhibit NO production by reducing iNOS protein expression in lipopolysaccharide-activated macrophages (Chung et al., 2003). In addition, oxyreveratrol (Figure 3) has a neuroprotective effects against neurotoxicity in cortical neurons induced by Aβ peptides (Niidome et al., 2007). Furthermore, decrease of NO and TNF-α in macrophages was demonstrated by a crude methanolic extract of mulberry leaves via reduction of activation of transcription factor NF-κB (Shibata et al., 2007). Another study has been proposed to elicit anti-inflammatory and neuroprotective effects useful in the treatment of brain ischemic injury (Wang et al., 2014). The mechanistic study showed that Mulberroside A exhibits anti-inflammatory and anti-apoptotic effects by decreasing the IL-6, IL-1β and TNF-α expression and inhibiting activation of LRR, NACHT, and PYD domains-containing protein 3 (NALP3), caspase-1, and NF-κB activation. All these findings indicate that oxyreveratrol is a potential candidate as a multifactorial neuroprotectant in AD (Wang et al., 2014).

#### Resveratrol

Resveratrol is a polyphenol derivative from *Vitis vinifera* (Table 2) belonging to the family of Vitaceae. It is reported to possess cardioprotective, anticarcinogenic, antioxidant, and anti-inflammatory activities in context to neurodegenerative disorders (Ma et al., 2014). Previous studies have revealed neuroprotective effect of resveratrol in rats having chronic unpredictable mild stress induced cognitive and emotional deficiencies (Yazir et al., 2015). Resveratrol (Figure 3) inhibits proinflammatory mediator's synthesis and release by modifying synthesis of eicosanoids, COX-2 and iNOS *via* inhibition of NF-κB (Alarcon De La Lastra and Villegas, 2005). Furthermore, in microglial cells resveratrol suppresses the level of expression of proinflammatory mediators TNF-α and NF-κB while promotes the IL-10 anti-inflammatory molecule (Song et al., 2014). These studies suggest its role against neurodegenerative diseases specially AD.

#### Apigenin derivatives

*Passiflora edulis* and *P. alata* (Table 2) produces flavonoids that improve behavioral performances in rats (Xu et al., 2013)*. Passiflora* has been used as a sedative, tranquilizer and natural anxiolytic agent (Barbosa et al., 2008). Flavonoids such as apigenin-8-*C*-β-digitoxopyranoside, luteolin-8-*C*-β-boivinopyranoside, and apigenin-8-*C*-β-boivinopyranoside (Figure 3) are the major phytochemicals contributing toward the effect of *Passiflora* (Xu et al., 2013). Recently apigenin has shown to reduce inflammation in case of LPS-induced microglial activation by inhibiting PGE_2_ and NO production by free radical scavenging (Xu et al., 2013). Furthermore, apigenin also decreases MAPK, JNK, p38, and ERK1/2 and also modulate neurite outgrowth induced by nerve growth factor (NGF) in PC12 cells (Xu et al., 2013). Additionally due to its BBB permeability, it serves as an effective phytochemical involved in the treatment of neurodegenerative diseases such as AD (Yang et al., 2014). Furthermore, apigenin and its *C*-glycosylated derivatives such as isovitexin and Vitexin possess activities against diabetes, Alzheimer's disease, and inflammatory activities (Choi et al., 2014). Due to its low intrinsic toxicity, apigenin has gained interest as a beneficial and health promoting agent. In LPS-induced macrophages the potential of apigenin and its derivatives were investigated against diabetes, Alzheimer's disease, and inflammation (Choi et al., 2014). The results showed relatively weak anti-AD and anti-diabetic potentials of apigenin as compared to anti-inflammatory properties that are shown by inhibiting iNOS, COX-2, and NO production as compared to its *C*-glycosylated derivatives (Choi et al., 2014). It can be assumed through these studies that apigenin might be useful against AD.

#### α-mangostin

*Garcinia mangostana* (Table 2), commonly called mangosteen is the fruit of a tropical evergreen tree native to Southeast Asia. Mangosteen possesses beneficial neuroprotective, anti-inflammatory and antioxidant effects. It consists of various polyphenolic xanthone derivatives such as α-mangostin. α-Mangostin (Figure 3) has shown to concentration-dependently attenuate Aβ-(1-40) or Aβ-(1-42) oligomers induced neurotoxocity, as observed in primary rat cortical neurons showing decrease in cell viability and impaired neurite outgrowth (Wang et al., 2012). Promising treatment and improved inflammation has been shown by mice fed with mangosteen supplemented diet (Huang et al., 2014). These studies suggest role of mangosteen in attenuation of AD.

#### Rosmarinic acid

*Melissa officinalis* (Table 2), commonly named as lemon balm, is used traditionally for its neuroprotective and antioxidant actions. It prevents neuronal cell death by scavenging free radicals (Pereira et al., 2009). Rosmarinic acid (Figure 3) from *Rosmarinus officinalis* enhances cholinergic activity in cell differentiation mediated by ERK1/2 suggesting neurotrophic effect in PC 12 cells (El Omri et al., 2010). Rosmarinic acid has show protection against neuronal damage induced by hypoxia resulting in proinflammatory cytokines such as TNF-α, IL-1β, and caspase 3 through suppression of hypoxia inducible factor-1α (HIF-1α) expression (Bayat et al., 2012). All these studies shows the rosmarinic acid and other derivatives from *M. officinalis* play an important role in improving cholinergic activity by the mechanisms underlying memory enhancing function. These studies suggest rosmarinic acid as a potential therapeutic agent against AD.

#### Quinic acid derivatives

*Pimpinella brachycarpa* (Table 2), belongs to family Umbelliferea. It is distributed in Asia, Europe, and Africa (Lee, 2003). Quinic acid derivatives were isolated from the MeOH extract of *P. brachycarpa* (Lee et al., 2013). Beneficial effects of quinic acid derivatives include antioxidant, anti-hepatitis B virus, carcinogenesis and anti-inflammatory effects (Wang et al., 2009). *Aster scaber*, from the family Asteraceae is another source of quinic acid derivatives containing (–)-3,5-dicaffeoylmucoquinic acid and (–)-4,5-dicaffeoylquinic acid (Hur et al., 2004). In a previous study, the anti-inflammatory activities of potential components of MeOH extract isolated from *P. brachycarpa* were evaluated on LPS-activated BV-2 microglial cells (Lee et al., 2013). MeOH extract obtained from aerial parts of *P. brachycarpa* were separated through column chromatography, which furnished fifteen quinic acid derivatives. Among these derivatives 1-***O***-trans-caffeoyl-5-***O***-7,3,5-***O***-trans-dicaffeoylquinic acid and 8-dihydro-7α-methoxycaffeoylquinic acid methyl ester (Figure 3) significantly inhibited inflammatory mediators in cell lines activated by LPS (Lee et al., 2013). Furthermore, *A. scaber* isolated quinic acid has been found to increase survival of C6 glioma cell upon tetrahydropalmatine (THP) induced toxicity because of free radical scavenging malondialdehyde (MDA) and superoxide dismutase (SOD) (Soh et al., 2003). It is also shown that (-)-3,5-dicaffeoylmucoquinic acid an extract from *A. scaber* affects neurite outgrowth by affecting PI3K and ERK1/2 *via* activation of TrkA signaling (Xiao et al., 2011). Thus, quinic acid extracted from *A. scaber* alleviate neurodegenerative diseases by protecting neurons from free radicals by enhancing the free radical scavenging system and attenuating pro-inflammatory responses by inhibiting iNOS expression (Soh et al., 2003). Therefore, this herb might be a potential candidate for AD due to significant anti-inflammatory activities.

#### Epigallocatechin-3-galate

Epigallocatechin-3-galate (EGCG) is a member of Theaceae family. This polyphenol is extracted from *Camellia sinensis* (Table 2) which is a natural green tea (Gramza-Michalowska and Regula, 2007). This herb is reported as beverage worldwide and is abundant in hilly areas of Asia (Gramza-Michalowska and Regula, 2007). Green tea catechins are brain permeable and in recent studies it has been ascribed to have neuroprotective actions as well (Singh et al., 2008). Several molecular biological roles are served by green tea catechins which include activation of antioxidant enzymes, protein survival genes, and kinase C, and APP processing (Levites et al., 2002). Green tea may also be involved in protection of neurons from Aβ-induced damages evidenced from several *in-vitro* studies (Bastianetto et al., 2006). Catechins have well documented anti-inflammatory properties. EGCG (Figure 3) inhibits NF-κB and MAPK in activation. It also inhibits the production of vascular endothelial growth factor IL-6, and IL-8 in U373MG cells of human astrocytoma (Kim et al., 2007). Anti-inflammatory activities of various cytokines are suppressed by EGCG, through inhibition of IL-1β and Aβ-induced COX-2 expression (Kim et al., 2007). Reportedly EGCG inhibits LPS-induced microglial activation, protection against neuronal injury mediated by inflammation (Li et al., 2004). EGCG increases NGF and CREB expression levels in APP/PS1 providing neuroprotection through ameliorating cognitive impairment in mice model, neuroprotection is also mediated by ERK1/2/ C-Raf by phosphorylating TrkA (Li et al., 2004). It also reduces activation of JNK2 and p75/CD reducing the expression levels of caspase 3 leading to reduction in levels of Aβ (1-40) and hippocampal APP expression (Liu et al., 2014). Prolonged consumption of green tea positively effects age-related neurodegeneration by increasing glutathione, potentiating, scavenging system, activating Bcl-2 protein and CREB levels and also boosting BDNF levels (Paulo Andrade and Assuncao, 2012). Chronic treatment has shown to improve spatial learning and memory due to the presence of catechins in green tea as shown in SAMP8 mice having impairment in memory and learning. Catechins through PKA/CREB signaling suppresses Aβ (1-42) level in the hippocampus and increases CaMKII and BDNF levels (Li et al., 2009).

Several animal models have been reported regarding the effects of green tea catechins on memory function. For instance, in Tg2576 mice reduction in cognitive impairment is shown by EGCG (Rezai-Zadeh et al., 2005). Intracerebroventricular injection of Aβ_1−40_ in rats showed less memory impairment when provided with high levels of green tea catechins especially EGCG with drinking water (Haque et al., 2008). Injecting tea catechins also ameliorates hippocampal neuronal damage and memory impairment in mouse model of cerebral ischemia (Lee et al., 2003). Thus, it is a better therapeutic approach to use green catechins, especially EGCG for the treatment of cognitive decline as in AD.

#### Curcumin

*Curcuma longa* (Table 2) from the family Zingiberaceae is commonly known as turmeric. It contains phenolic constituents which includes curcumin, bisdemethoxycurcumin, and demethoxycurcumin (Akbik et al., 2014). It is used in flavoring food preparations commonly, also due to its good medicinal properties it is used for the treatment of coughs, biliary disorders, hepatic disorder, diabetic ulcer, rheumatism and sinusitis (Akbik et al., 2014). Curcumin (Figure 3) exhibits anti-inflammatory and anti-oxidant properties (Aggarwal and Harikumar, 2009). In rat model of olfactory-bulb-ablation, curcumin has been found to suppress the levels of TNF-α and caspase 3, the neuroinflammatory mediators by increasing the BDNF levels (Rinwa et al., 2013). Studies have revealed the involvement of curcumin in regulation of CREB and BDNF levels in D-galactose-induced memory and learning deficits in mouse models (Nam et al., 2014). Curcumin also treated chronic unpredictable stress induced cognitive deficiency in rats by recovering the ERK1/2 and BDNF levels in hippocampus (Liu et al., 2014). In cisplatin-treated PC12 cells curcumin affected expression level of p53 and inhibition of neurodifferentiation was reduced (Mendonça et al., 2013). Apoptosis induced by β-amyloid was attenuated by curcumin *via* inhibition of NF-κB activation (Kuner et al., 1998). Moreover cognitive decline induced by Aβ (1-42) in rats was treated by nanoencapsulated curcumin by increasing the levels of BDNF and regulating signaling of Akt/GSK-3 β in microglial cells and astrocytes leading to modulation of tau hyperphosphorylation along with an increase in synaptophysin levels in hippocampus (Hoppe et al., 2013). Furthermore, lead acetate-induced oxidative stress in rats can be regulated by curcumin has shown to increase the levels of antioxidants (Hosseinzadeh et al., 2013). Through various models of neurodegeneration, curcumin has shown to be involved in neuroprotection and recovery of memory and learning by increasing the BDNF levels and exerting anti-neuroinflammatory and anti-oxidant properties, which speculates the beneficial role of curcumin in the treatment of AD.

#### 6-shogaol

*Zingiber officinale* (Table 2) from the family Zingiberaceae is commonly known as ginger. It contains a phenolic phytochemical, 6-shogaol, a compound used as a culinary spice and traditionally used for centuries in Siddha, Indian, Unani, Chinese, Tibetan, and Arabic medicinal practices (Haniadka et al., 2012). A wide variety of phytochemicals are derived from ginger which includes 6-shogaol, 6-gingerol, 8-gingerol, and 10-gingerol, showing positive effects in motion sickness, vomiting, and nausea (Palatty et al., 2013). It also has reported anti-inflammatory, antioxidant, and anti-cancer activity (Shim et al., 2011). Many studies have shown potent activity of 6-Shogaol (Figure 3) against AD, enhancing memory, inhibiting inflammation and boosting antioxidant system (Moon et al., 2014). In scopolamine–and Aβ (1-42)-induced dementia mice models, it improved cognitive impairment by inhibiting inflammatory mediators and increasing NGF levels, and postsynaptic proteins in hippocampus (Moon et al., 2014). On LPS-treated BV2 and primary microglial cells, 6-shagaol has a beneficial effect by inhibiting COX-2, PGE_2_, NO, iNOS, P38 MAPK, IL-1β, TNF-α, and NF-κB (Ha et al., 2012). Additionally 6-shagaol exhibits neuroprotective effect by increasing the choline acetyltransferase, BDNF expression, and reducing ROS release through TrkB-mediated signaling in H_2_O_2_-treated HT22 hippocampal neuronal cells (Shim and Kwon, 2012). Further studies also assessed the neuroprotective role of 6-shagaol against LPS-induced inflammation (Shim et al., 2011). It suppressed the pro-inflammatory cytokines release and decreases the level of iNOS, NF-κB, and COX-2 in astrocytes treated with LPS (Shim et al., 2011). These studies revealed the valuable phytotherapeutic property of 6-shagaol for the treatment of neurodegenerative diseases like AD.

#### Naringenin

Naringenin a potent flavonoid is found to be abundant in citrus fruits such as grapefruit (*Cirus paradise*) and oranges (*Citrus sinensis*) (Table 2). In male SD rats, naringenin (Figure 3) showed protection against focal cerebral ischemia by downregulating NF-κB, nucleotide oligomerization domain protein 2 (NOD2), mitochondrial membrane potential (MMP), receptor-interacting protein 2 (RIP2) and upregulating claudin-5 (Bai et al., 2014). Recent studies have shown the protective ability of naringenin in rat models of focal cerebral I/R injury by prevention of inflammatory damage mediated by NF-κB and oxidative stress (Raza et al., 2013). It upregulates antioxidant status, decreases myeloperoxidases, nitric oxide, and cytokines, also downregulates the expression level of NF-κB (Raza et al., 2013). Naringenin also prevented 6-hydroxydopamine (6-OHDA)-induced neurotoxicity by activating Nrf2/ARE signaling (Lou et al., 2014). Through these studies naringenin can be speculated as a potential agent against AD.

### Neuroprotective effect of terpenoid-derived phytochemicals in Alzheimer's disease

#### Ligraminol E4-*o*-β-d-xyloside

*Abies holophylla* is a member of the family Pinaceae, commonly named as the Needle Fir or Manchurian Fir. *A. holophylla* (Table 3) is present in evergreen forests of Korea, Russia and China, and (Kim et al., 2013). Seventeen different lignans were found in the ethanol extract of *A. holophylla*. In murine microglial cells, LPS-induced production of NO was potently inhibited by lignans and were also found to increase NGF levels in C6 glial cell cultures (Kim et al., 2013). Two novel sesquiterpenes, such as (8R,9S,7′S,8′R)-4,4′,7′-trihydroxy-3,3′,9-trimethoxy-9,9′-epoxylignan and ligraminol E4-***O***-β-**d**-xyloside (Figure 3) were found in *A. holophylla* exhibited significant inhibition of NO expression in activated microglial cells (Xia et al., 2012). Abies spp. has shown to have protective effect due to potently reducing inflammation in brain cells (Xia et al., 2012). Therefore, this herb due to its significant anti-inflammatory properties in brain can be used as a potential therapeutic agent in AD.

**Table 3:** Terpenoid-derived phytochemicals that affect Alzheimer's disease.

**Plant source**	**Phytochemicals**	**Pharmacological effects**	**Medicinal use**	**References**
**TERPENOID-DERIVED PHYTOCHEMICALS**				
*Abies holophylla*	Ligraminol E4-*O*-β-d-xyloside	Inhibits NO production	Neurodegenerative disease, and neuroinflammatory	Xia et al., 2012
*Ginkgo biloba*	Ginkgolide B	Suppresses NF-κB, PI3K/Akt pathway, upregulates anti-apoptotic proteins expression, induces BDNF secretion and reduces ROS, LDH and caspase3	Dementia, neuroprotective, antioxidant and neuroinflammation	Xiao et al., 2010; Nabavi et al., 2015
*Melia toosendan*	Limonoid, 1α,3α-dihydroxyl-7α- tigloyloxy-12α-ethoxylnimbolinin & 12-*O*-ethyl-1-deacetyl-nimbolinin B	Activates PKA/ERK1/2-mediated neurite outgrowth, induces NGF secretion, and decreases LDH activity	Neurodegenerative disease, neuroprotective, and neuroinflammatory.	Roy and Saraf, 2006
*Panax ginseng*	Ginsenoside Rg3	Activates cAMP/MAPK & Trk-mediated Neuritogenesis and inhibits TNF-α, NF-κB, IL-1β, and iNOS	Neuroinflammation, neurodegenerative disease, and neuroprotection	Joo et al., 2008

#### Ginsenoside Rg3

*Panax ginseng* (Table 3) is a member of family the Araliaceae. It is a perennial plant mostly found in Korea, Vietnam China, Russia, and Japan (Chung et al., 2011). Triterpenoid saponin, ginsenoside Rg3 and steroidal saponins are the main constituents of *P. ginseng* (Chung et al., 2011). Ginsenoside Rg3 (Figure 3) and other derivatives crosses BBB to a sufficient degree (Wang et al., 2006b). Previous studies have shown that extract of red ginseng is therapeutically effective against neurodegenerative diseases through control of apoptotic-and inflammatory-related events (Joo et al., 2008). It also reduces Aβ42-induced toxicity in BV-2 cells by inhibiting TNF-α, NF-κB, IL-1β, and iNOS (Joo et al., 2008). In addition, ginsenoside Rg3 potently increases neuritogenesis and cholinergic markers through targeting NGF-TrkA signaling (Kim et al., 2014). Therefore, *P.ginseng* strongly reduces neuroinflammatory cytokines and also induces immune cells to ingest the oligomeric plaques formed in AD.

#### Ginkgolide

*Ginkgo biloba*, a member of family Ginkgoaceae, traditionally used in Chinese medicine. For thousands of years it has been used for the treatment of neurological diseases such as neurosensory disorders and dementia associated with neurodegeneration (Fang et al., 2010). Extensive studies have revealed that *G. biloba* (Table 3) has promising effects against AD, PD, ischemic stroke (Fang et al., 2010). Ginkogolide B (Figure 3) is a potent neuroprotective compound used in treatment of neurodegenerative diseases is extracted from *G.biloba*. it is reported to have the ability to cross BBB, more particularly in ischemic conditions (Fang et al., 2010). Numerous studies have reported the protective effect of ginkgolide B against ischemic stroke by increasing sirt1 (silent mating type information regulation 2 homolog-1) expression, suppresses NF-κB, inhibits PI3K/Akt pathway and TLR-4/ NF-κB, up-regulates heme oxygenase 1, anti-apoptotic protein expression, and erythropoietin secretion (Nabavi et al., 2015). It also inhibited pro-apoptotic protein expression, and improves endothelial NO synthesis (Nabavi et al., 2015). It has also been shown that *G. biloba* possesses antioxidant activity by attenuating endoplasmic reticulum (ER) and mitochondrial dysfunctions induced by bupivacaine and ROS (Li et al., 2013). This activity attained through reduction in mitochondrial toxicity *via* reduction in the protein levels of Htra2, caspase 3, caspase 12 and the mRNA in cell lines of human neuroblastoma (Li et al., 2013). In Aβ25-35-treated neuron cultures of primary hippocampus, ginkogolide B was shown to increase BDNF expression levels exerting neuroprotection by reducing caspase 3, the K^+^ ion level and lactate dehydrogenase (LDH) (Xiao et al., 2010). Extracts of *G.biloba* have shown to exhibit neuronal protection by inducing Trk-mediated axonal growth and suppressing apoptotic factors and ROS production (Xiao et al., 2010). On the basis of these studies it can be assumed that ginkolide can be used in amelioration of AD.

#### Limonoids

*Melia toosendan*, belongs to the family Meliaceae containing triterpenoids. Limonoids (Figure 3) have shown to have antibacterial, neuroprotective effects, and anti-carcinogenic properties (Roy and Saraf, 2006). *M. toosendan* contains 12-*O*-ethyl-1-deacetyl-nimbolinin B and 1α, 3α-dihydroxyl-7α-tigloyloxy-12α-ethoxylnimbolinin fruit extracts, which dose-dependently induce the neuronal differentiation by causing increase in neurite outgrowth in macrophages in rats along with the increase in secretion of NGF (Zhang et al., 2013). *M. toosendan* contains many neuroactive compounds involved in induction of neurite outgrowth in PC12 cells exposed to PKA inhibitors (Jowie and Ip, 2004). These studies suggest a significant potential of limonoids extracted from *M. toosendan* aganist neurodegenerative diseases like AD (Table 3).

### Neuroprotective effect of alkaloidal phytochemicals in Alzheimer's disease

#### Berberine

*Coptis chinensis*, belongs to the Ranunculaceae family. The major component of the plant is berberine (Figure 3) is an isoquinoline alkaloid (Asai et al., 2007). In China it is widely used as a herbal medicine for treatment of liver disease, microbial infection and skin inflammation since decades (Asai et al., 2007). Various studies have reported the neuroprotective effects of berberine using neurodegenerative disease models (Jia et al., 2012). In scopolamine-induced memory impairments berberine reduces production of the proinflammatory cytokines TNF-α, COX-2, and IL-1β and restores levels of CREB and BDNF as well as reduces the latency of escape in rats (Lee et al., 2012). In Aβ-induced neuroinflammation in murine primary microglia and cultured BV2 cells, pretreatment with berberine prevented MCP-1 and IL-6 productions and also downregulated the expression of COX-2 and iNOS expression (Jia et al., 2012). This anti-neuroinflammatory potential of berberine suggests its role in attenuation of AD (Table 4).

**Table 4:** Alkaloidal phytochemicals that affect Alzheimer's disease.

**Plant source**	**Phytochemicals**	**Pharmacological effects**	**Medicinal use**	**References**
**ALKALOIDAL PHYTOCHEMICALS**				
*Coptis chinensis*	Berberine	Activates AKT/GSK-3β/Nrf2-mediated regulation, induces NGF and BDNF secretion, and inhibits COX-2, TNF-α, NF-κB, IL-1β, and iNOS levels	neuroinflammation, and neuroprotection	Jia et al., 2012; Lee et al., 2012
*Galanthus* Sp.	Galantamine	AChE inhibitor, inhibits IL-1β generation and microglial accumulation, increases antioxidant enzymes	Neurodegenerative diseases, neuroprotection, anti-inflammatory and anti-oxidant	Furukawa et al., 2014
*Huperzia serrata*	Huperzine A	Increases secretion of BDNF, SOD, GST and catalse, inhibits AChE, NF-κB, TNF-α, and caspase-3	Antioxidant, anti-inflammatory, and neuroprotection	Mao et al., 2014
*Sophora flavescens*	Sophocarpidine	Decreases expression of interleukin-1β	Antineuroinflammatory, Anti Alzheimer's disease	Ni et al., 2006

#### Huperzine A

*Huperzia serrata* contains a sesquiterpene alkaloid i.e., huperzine A (Table 4). For decades it has been used in Chinese medicines as a reversible acetylcholinesterase (AChE) inhibitor (Zu Zhu et al., 2004). It exerted neuroprotective effect toward AD by promoting anti-apoptotic protein expression, inhibiting AChE, and NGF, resulting in alteration of Aβ peptide processing and reduction of oxidative stress (Zhang and Tang, 2006). Huperzine A (Figure 3) reduced the D-galactose-induced inflammatory loss in rat hippocampus through NF-κB and inhibiting neurovascular damage and BBB impairment (Ruan et al., 2014). (Ruan et al., 2014). In mouse models of reperfusion injury and transient cerebral ischemia, memory impairment was recovered by increasing the levels of BDNF, TGF-β and NGF *via* MAPK/ERK-mediated neuroprotection (Wang et al., 2006a). In SHSY5Y neuroblastoma cells, treatment with huperzine A caused activation of p75_NTR_ and TrkA receptors that resulted increase in MAP/ERK signaling reversing the reduction in NGF level in H_2_O_2_-induced oxidative stress model (Tang et al., 2005). In streptozotocin-induced diabetic rats, huperzine A has shown to attenuate cognitive defects by increasing the levels of glutathione peroxidase, BDNF, catalase, and SOD while simultaneously inhibiting, CAT, MDA, TNF-α, NF-κB, AChE IL-6, and caspase-3 (Mao et al., 2014). Huperzine A also prevented cognitive decline and brain damage in neonatal rats having hypoxia-ischemia induced brain injury (Wang et al., 2008). In rats with transient focal cerebral ischemia huperzine A is involved in protection through cholinergic anti-inflammatory pathway (Wang et al., 2008). This anti-inflammatory potential of huperzine A suggests that this herb can be used in the treatment and prevention of AD.

#### Galantamine

Galantamine is an alkaloid derivative of *Galanthus* species (Table 4), belonging to the family of Amaryllis. It displays therapeutic benefits against AD (Furukawa et al., 2014). However, it was reported therapy against mild AD, due to reversible antagonism of acetylcholinesterase (AChE) by degrading acetylcholine (Jackisch et al., 2009). Due to its hydrophobic nature, galantamine crosses blood brain barrier, similar to AChE inhibitor i.e., physostigmine (Jackisch et al., 2009). Galantamine (Figure 3) has also been reported to decrease brain injury in rats induced by hypoxia-ischemia via inhibition of IL-1β generation and microglial accumulation (Furukawa et al., 2014). In preclinical studies, galantamine has also been shown to increase antioxidant enzymes offering neuroprotection reducing neurodegeneration caused by ROS induced by Aβ in AD (Jackisch et al., 2009).

#### Sophocarpidine

Sohocarpidine is isolated from roots of *Sophora flavescens*. Evidences suggests sophocarpidine (Figure 3) decreases expression of interleukin-1β in cerebral cortex and hippocampus and in AD rat model it also alleviates injury in mitochondria of neuronal cells, established by ibotenic acid injection into the hippocampus (Ni et al., 2006). The anti-AD effect of sophocarpidine is due to the mitigation of inflammation by suppressed release of inflammatory cytokines in the brain (Table 4) and thus improving the status of neuronal cells injury and reduction in apoptosis of neuron (Ni et al., 2006).

## Conclusion

Despite the increasing prevalence of AD worldwide, there is still no cure of this disease. Extensive efforts have been made by researchers to find its solution, however, currently available therapeutic agents unfortunately target only the symptoms not the exact cause of this disease. Various hypotheses have been put forward to explain the etiology and pathology of AD in order to deal with the devastating cognitive decline observed in patients with AD. The multi target approach provides more therapeutic value. In order to extend these observations, anti-inflammatory and antioxidant targets have been focused to evaluate the protective effects against Aβ-induced neurotoxicity. Early trail with NSAIDs such as indometacin and other drugs from this group suggested reduced cognitive decline but could not replicate the results on large scale trails which seemed to be unsuccessful (Aisen et al., 2003; de Jong et al., 2008). Likewise, randomized trials with other anti-inflammatory drugs such as simvastatin (Simons et al., 2002), hydroxychloroquine (Van Gool et al., 2001), prednisone (Aisen et al., 2000), aspirin (Bentham et al., 2008), atorvastatin (Sparks et al., 2005), and rosiglitazone (Harrington et al., 2011) failed to show significant clinical changes in patients with primary cognitive decline. However, NSAIDs like naproxen and celecoxib initially showed a detrimental effect, a longer-term follow up of these patients suggested that naproxen provided protection in patients who had been asymptomatic at baseline (Breitner et al., 2011). This indicates that timing and choice of specific anti-inflammatory will provide better results (Breitner et al., 2011). There is an empirical evidence of prevention and treatment of AD by natural products (Howes et al., 2003). Plant rich diet like fruits, grains, and vegetables are correlated with healthy aging and well-being with reduced risk of AD (Howes et al., 2003). Their potential application in cure and prevention is supported by various experimental studies (Essa et al., 2012). Pharmacological basis for the use of natural products is due to their safety and efficacy as compared to other investigational drugs. Therefore, they might be used as an alternative to conventional treatments. Evidence from the previous literature suggests that phytochemicals that affect anti-inflammatory and downstream signaling targets have shown promising results in *in-vivo* and *in-vitro* studies, and therefore could be used to prevent the AD progression. The review comprehensively discussed the protective role of phytochemicals and their mechanism of action and demonstrated a safer approach toward the protection of neuronal damage caused by inflammation and oxidative stress in AD. Phytochemicals are promising therapeutic agents against neurodegenerative diseases due to their anti-inflammatory, antioxidant as well as anticholinesterase properties. This review focused on the neuroinflammation driven neurodegeneration particularly in AD and the importance of phytochemicals in its prevention and cure by targeting various molecular pathways involved in regulating neurodegenerative diseases.

## Future perspective

The numbers of neurodegenerative cases are increasing with the increase in aging in population. This might be due to the increase in chronic inflammatory diseases. Understanding the mechanism of neuroinflammation and neurodegeneration relating to systemic inflammation would be an interesting area for future research. There is still an underrepresentation of phytochemicals that regulate neurodegenerative diseases in preclinical *in vivo* studies. The possible preclinical stages could provide the best window for therapeutic or preventive approaches toward the systemic and central role of inflammation in neurodegenerative diseases.

## Author contributions

BS and WD equally to this work in study design and writing of the manuscript. HA perform interpretation and critical review and drafting of the manuscript. SK and YK substantial contribution to the concept and designing of study and revision of manuscript thoroughly. All authors listed have made a substantial, direct, and intellectual contribution to the work. They also read and approved the final manuscript.

### Conflict of interest statement

The authors declare that the research was conducted in the absence of any commercial or financial relationships that could be construed as a potential conflict of interest.
